# Characterization of the complete mitochondrial genome of *Eriocheir leptognathus* with phylogenetic analysis

**DOI:** 10.1080/23802359.2018.1535858

**Published:** 2018-11-25

**Authors:** YiMing Yuan, YanLong He, ShouHai Liu, Xiao Ji, YuTao Qin, XiaoBo Wang

**Affiliations:** a East China Sea Environmental Monitoring Center of State Oceanic Administration, Shanghai, China;; b Key Laboratory of Integrated Monitoring and Applied Technology for Marine Harmful Algal Blooms, SOA, Shanghai, China

**Keywords:** *Eriocheir leptognathus*, complete mitochondrial genome, phylogenetic analysis, comparative mitogenomic analysis

## Abstract

*Eriocheir leptognathus* is a dominant species in the Yangtze River estuary. In this study, we first determined the complete mitochondrial genome (mitogenome) of *E. leptognathus*. The mitogenome is 16,143 bp in length, consisting of 13 protein-coding genes (PCGs), 22 transfer RNA (tRNA) genes, two ribosomal RNA (rRNA) genes, and one non-coding control region. Initiation codons ATG and ATT were identified in eight and four PCGs, respectively, while stop codons TAA or TAG were found in eleven genes except for two genes which use incomplete stop codon T–. The phylogenetic analysis indicated that three species (*E. hepuensis*, *E.japonica*, *E. sinensis*) and *E. leptognathus* are very closely related. The complete mitogenome of *E. leptognathus* can provide population genetics information to further explore the taxonomic status of this species.

*Eriocheir leptognathus* is widely distributed in brackish waters from Fujian province to the eastern coast of the Korea peninsula (Wang and Chen 2003). According to a previous investigation, *E. leptognathus* was identified as a dominant species in the Yangtze River estuary (Chao et al. 2015). Its larval morphological characteristics, breeding season and larval living environment are similar to those of *E. sinensis* (Wang and Chen 2003). Mitochondrial DNA has many characteristics, for instance, maternal inheritance, relatively high evolutionary rate, and conserved gene components (Moore 2010; Yan et al. 2018), which make it highly useful in phylogenetic relationships analysis.

*Eriocheir leptognathus* was collected from Chongming, China (121°47.180′, 31°12.966′). The specimens now were stored in East China Sea Environmental Monitoring Center. Total mitochondrial genomic DNA was sequenced and annotated. According to the mtDNA sequences, protein-coding genes (PCGs) were identified using BLAST search in NCBI. And tRNA genes were identified using the tRNAscan-SE search server (Schattner et al. 2005).

The complete mitochondrial genome of *E. leptognathus* (Genbank accession MH593561) is 16,143 bp in length and contains 13 protein-coding genes (PCGs), 22 tRNA genes, two rRNA genes, and one non-coding control region (D-loop). Most mitochondrial genes are encoded on the H-strand except for four PCGs and six tRNA genes. The overall base composition is A (35.60%), T(39.04%), C(15.29%), and G(10.07%), with an AT content of 74.65%, which is consistent with most mitochondrial genomes. For the 13 PCGs, the lengths range from 162–1731 bp. ATG is the initiation codon of eight PCGs (*CYTB*, *ATP 8*, *COX1*, *COX2*, *COX3*, *ND4*, *ND4L*, and *ND5*), ATT was the initiation codon of four PCGs (*ATP6*, *ND2*, *ND3*, *ND6*), and *ND1* uses ATA as the initiation codon. Most of them use TAA as the stop codon, except *ND2* which uses an uncommon TAG stop codon, and *CYTB* and *COX1,* which uses an incomplete stop codon T–. The 12S rRNA (882 bp) and 16S rRNA (1315 bp) are located in the positions between *tRNA-Leu* and *ND5*. And the 22 tRNA genes range from 63 bp (*tRNA-Cys* and *tRNA-Arg*) to 73 bp (*tRNA-Val*). The length of non-coding region or control region is 802 bp. The A-T content of this region is high, up to 83.04%, which is thought to be with the transcription and replication of the mitogenome (Clayton 1992). In addition, we detected five instances of microsatellite-like (AT) _n_ element, and its repeat counts from 5 to 9. The microsatellite-like (AT) _n_ is a common element in the D-loop region (Tang et al. 2018).

The phylogenetic relationship was recovered based on 13 PCGs. The phylogenetic tree in Figure 1 shows the relationship between *E. leptognathus* and other 14 crabs. We can clearly find that three species (*E. hepuensis*, *E.japonica*, *E. sinensis*) (Wang et al. 2016) are closest to *E. leptognathus*. The phylogenetic tree provides a reference for understanding the taxonomic status.

**Figure 1.**
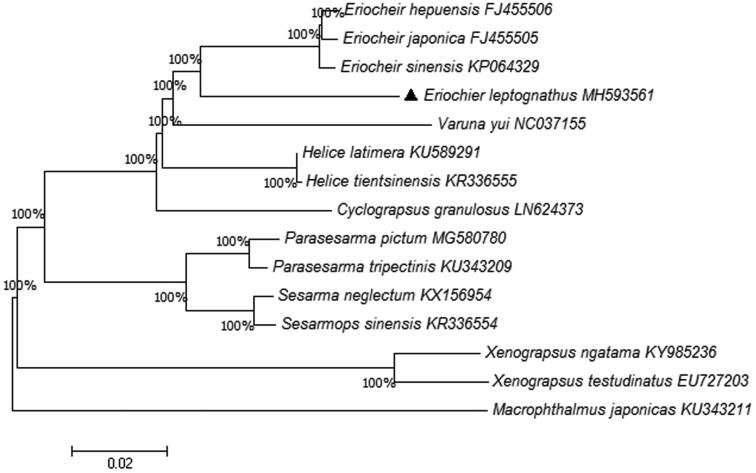
Phylogenetic tree derived from Neighbor Joining based on 13 protein coding genes. Fourteen mitogenome sequences were obtained from GenBank and included in the tree with their accession numbers. The GenBank accession numbers are indicated after the scientific name. The percentage at each node is the bootstrap probability.
